# Supplementary material for: Modular architecture confers robustness to damage and facilitates recovery in spiking neural networks modeling in vitro neurons
Source: Front Neurosci. 2025 Jun 19;19:1570783. doi: 10.3389/fnins.2025.1570783 (PMC12224873; doi:10.3389/fnins.2025.1570783)
Supplement: Supplementary file 1 [file Data_Sheet_1.pdf]

Supplementary Material

1 Supplementary Figures

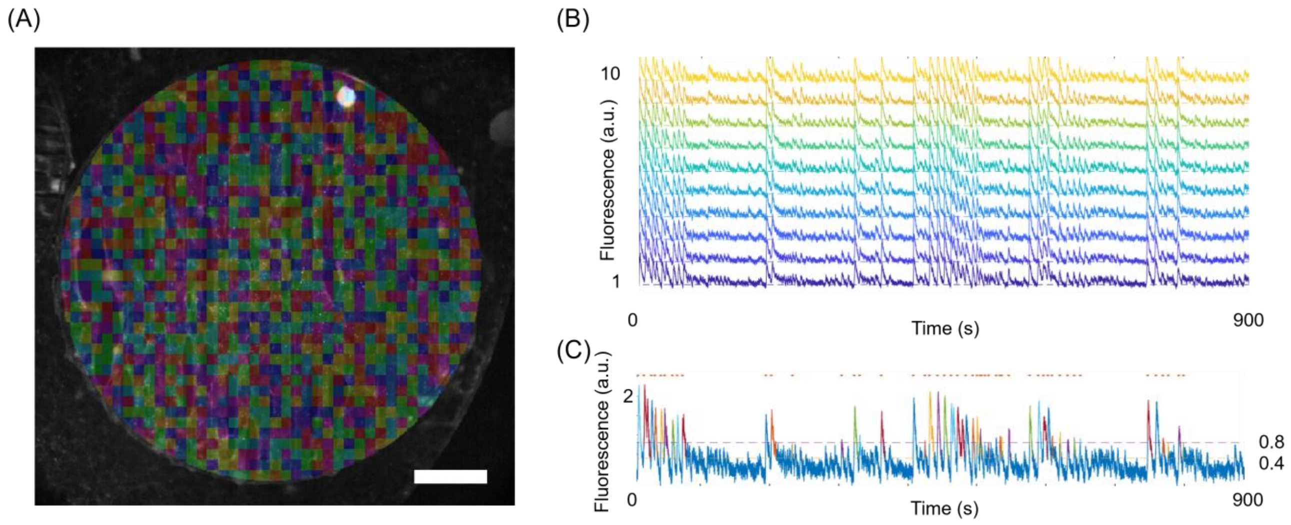

Supplementary Figure S1. Spike detection from in vitro neuronal recordings. (A) Selection of Region of Interest (ROI). A 150 µm × 150 µm square region of calcium intensity data was designated as the ROI for neurons cultured on 6 mm diameter PDMS disks. Approximately 1,280 ROIs were selected. Scale bar is 1 mm. **(B)** Representative mean fluorescence intensity data in the ROIs. Ten representative examples were plotted. **(C)** Spike detection using a Schmitt trigger (lower threshold = 0.4, upper threshold = 0.8). Spikes are indicated by orange dots in the plot.

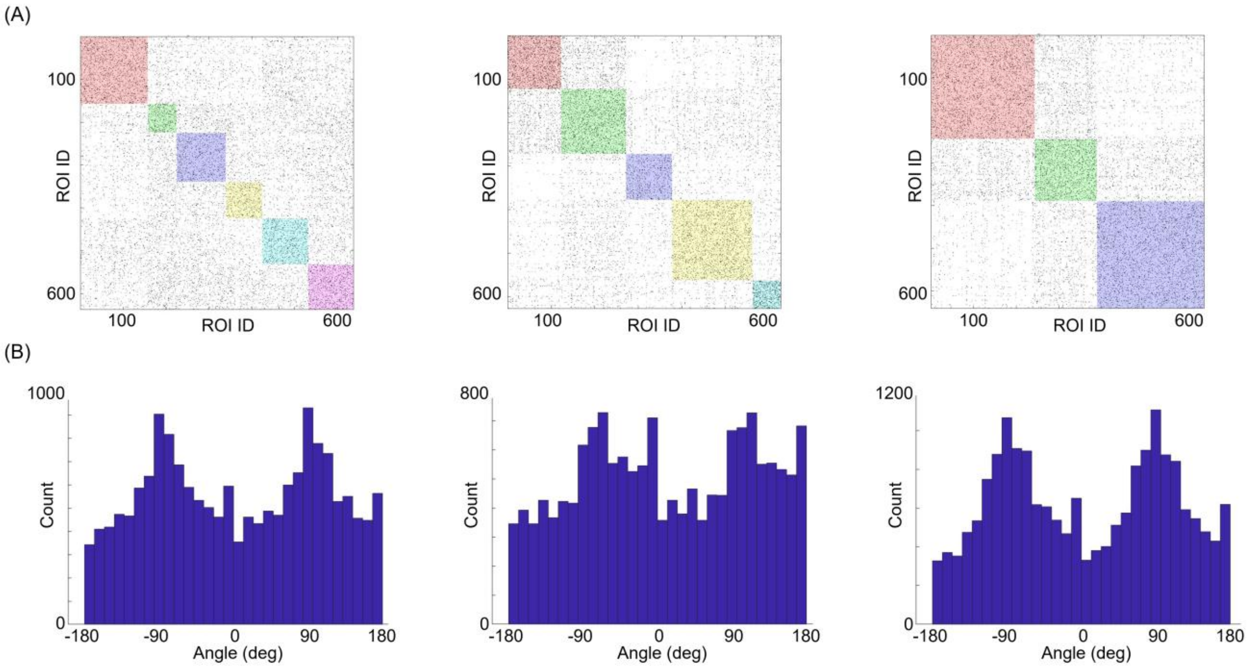

Supplementary Figure S2. Reorganization of effective connectivity and connection geometry following injury in vitro. (A) Effective connectivity maps estimated by transfer entropy before (left), immediately after (center), and 24 hours after (right) the injury. The colors represent modules when modularity is maximized. To enhance visualization, the analysis includes half the total ROIs. **(B)** Histogram of connection angles. Before the injury, sharp peaks appear at -90° and 90° (left), reflecting the dominance of vertical connections. These peaks are blunted after injury (center), but reappear 24 hours later (right).

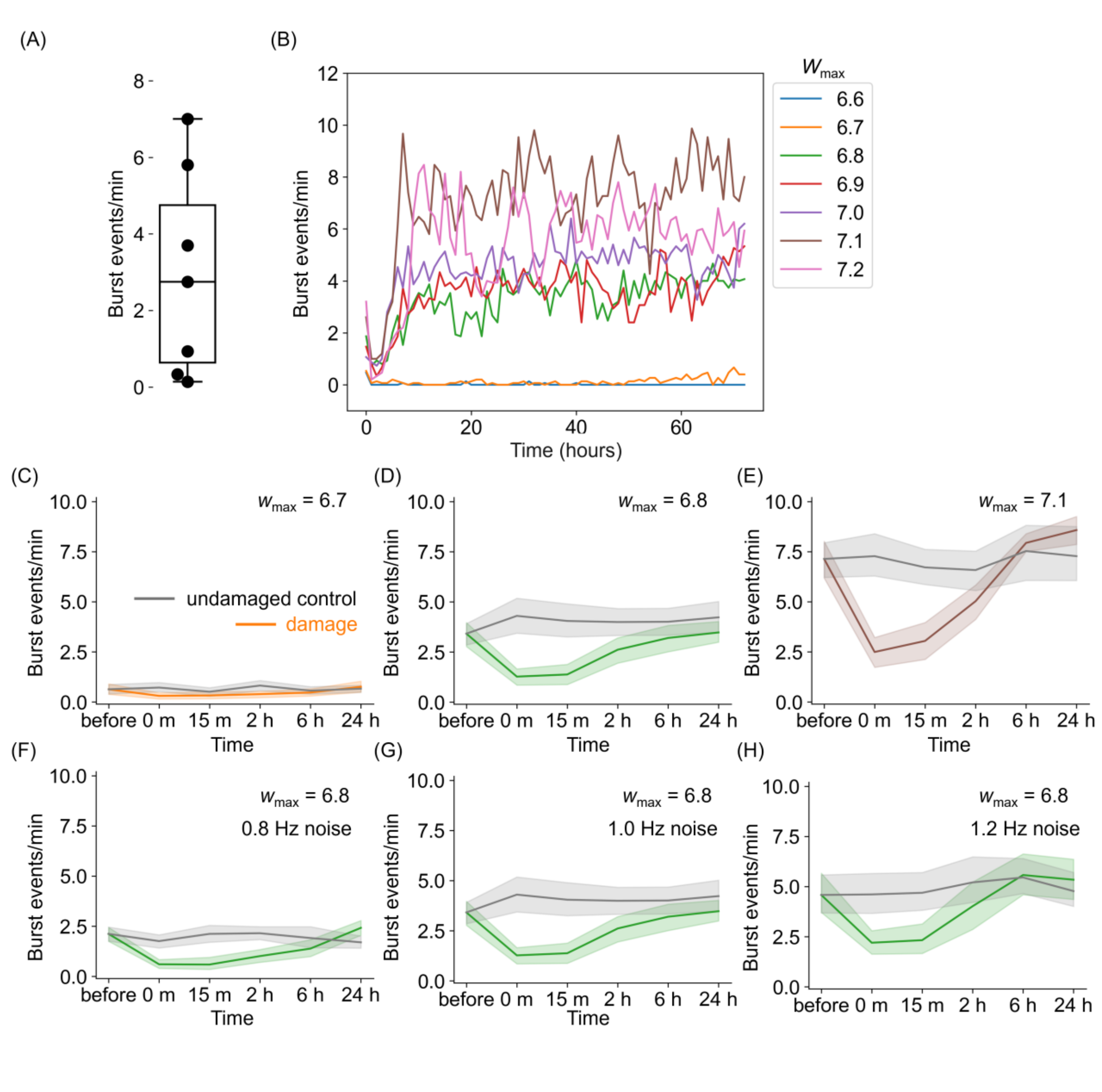

Supplementary Figure S3. Parameter exploration to reproduce in vitro burst event rates in simulation. (A) Burst event rate per minute in cultured neuronal networks before injury (n = 7). **(B)** Detailed evolution of the burst event rate in simulations for different maximum synaptic weights (w_{max}) in the pre-damage condition, with identical spontaneous noise frequency. In general, the higher w_{max}, the higher the frequency of bursting. The event rate after damage initially increases due to STDP but stabilizes after approximately 30 simulated hours. **(C−E)** Repetition-averaged time course of burst event rate for gradually higher w_{max} and constant noise frequency of 1 Hz, comparing damaged and control undamaged conditions (gray), with (C) w_{max}=6.7, (D) w_{max}=6.8, and (E) w_{max}=7.1. Solid lines represent mean values of n = 10 simulations, and shaded areas indicate 95% confidence intervals. **(F−G)** Time course of burst event rate for gradually higher spontaneous noise frequency and constant w_{max}. (F) 0.8 Hz, (G) 1.0 Hz, and (H) 1.2 Hz. The (G) is identical to (D)**.**

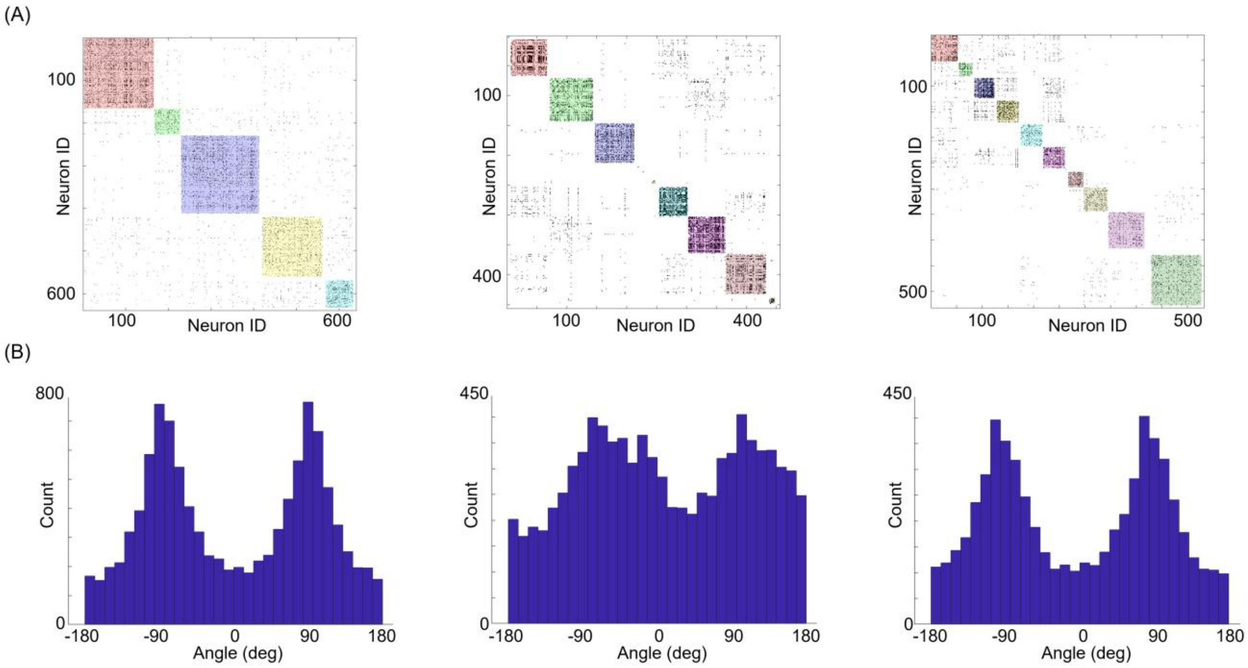

Supplementary Figure S4. Reorganization of effective connectivity and connection geometry following injury in silico. (A) Effective connectivity in the spiking neural network model before (left), immediately after (center), and 24 hours after (right) the injury. The colors represent modules when modularity is maximized. To improve visualization, the analysis includes one-quarter of the total neurons. **(B)** Histogram of connection angles. As in the culture experiment, sharp peaks at -90° and 90° are observed before the injury (left). These peaks blunted after the injury (center) but reappear after 24 hours (right)

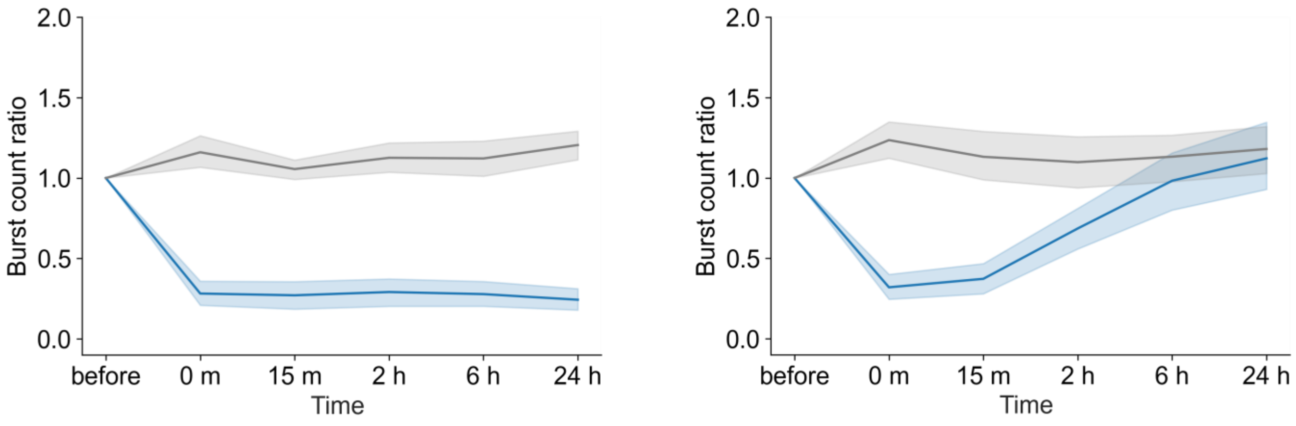

Supplementary Figure S5. Effect of STDP on post-injury recovery of burst activity in silico. Time course of burst event rate without STDP (left) and with STDP (right). The blue line shows damaged samples, and the grey line shows undamaged controls. Shaded areas represent the 95% confidence interval. In undamaged samples, burst activity remained stable over time. Damage initially reduced activity, followed by sustained low activity without STDP. However, with STDP, the activity recovered to a level comparable to the undamaged samples within 24 hours.

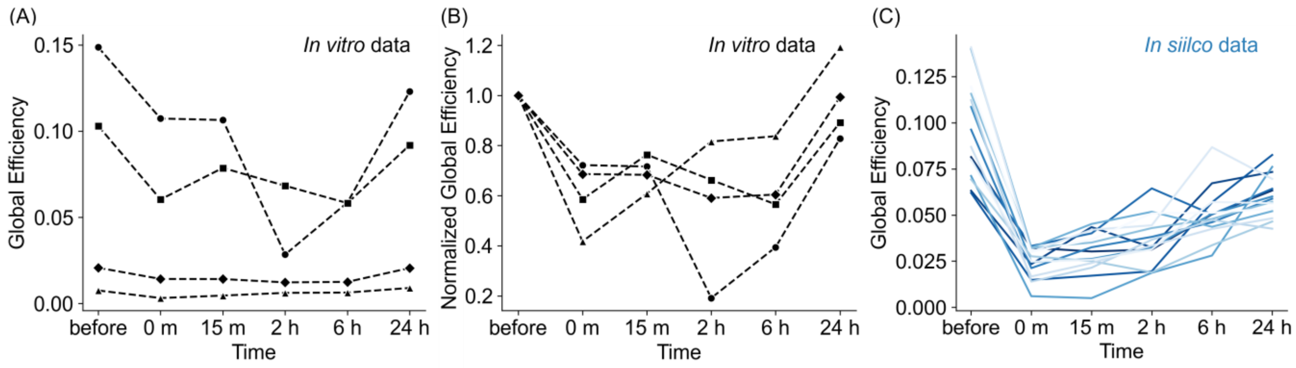

Supplementary Figure S6. Changes in global efficiency following injury in cultured and simulated neuronal networks. (A) Evolution of the global efficiency in cultured neurons before damage and during recovery, computed from TE-inferred weighted effective connectivity matrices. Each dot represents an individual sample across a full sequence of damage and recovery, with $n = 4$ sequences. **(B)** The same data after normalizing each sequence by the value of the global efficiency before damage. **(C)** Equivalent analysis for TE-inferred effective connectivity networks in numerical simulations, with $n = 15$ sequences of damage, illustrating the strong qualitative similarity of the *in silico* results with the *in vitro* ones.

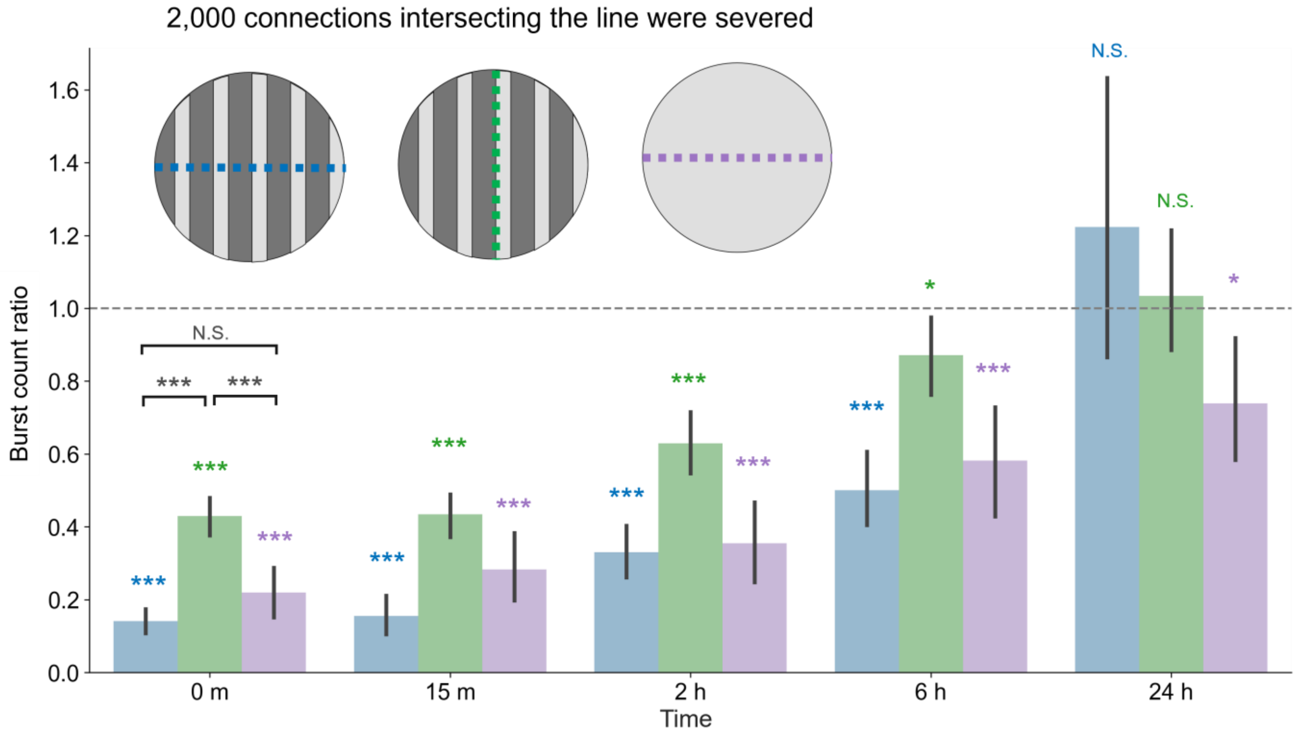

Supplementary Figure S7. Effect of damage configuration on burst activity with equal disconnection count in silico. Burst frequency changes for scenarios with the same number of connections to be disconnected. For each scenario, the color bar shows the ratio of burst frequency between the post- and pre-injury conditions. The bar height indicates the mean, and error bars indicate 95% confidence intervals. Color asterisks indicate a two-sided 1-sample *t*-test (*$p < 0.05$; **$p < 0.01$; ***$p < 0.001$; N.S., no significance; $n = 15$, $df = 14$). Asterisks in black correspond to a two-sided unpaired *t*-test (**$p < 0.01$; ***$p < 0.001$; N.S., no significance; $n = 15$, $df = 13$). Even when the number of disconnections was common, the effects of the direction of damage and the background module structure were consistent with the results in Figure 4B.

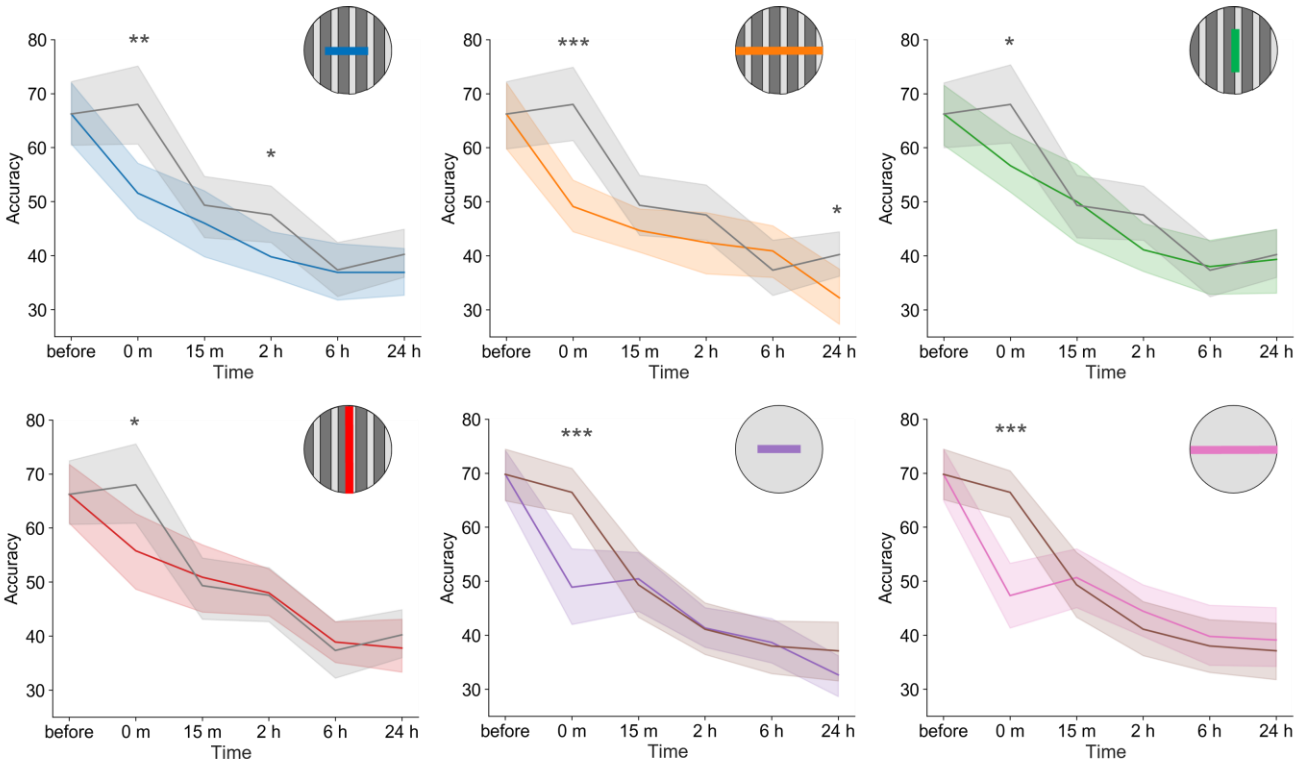

Supplementary Figure S8. Reservoir computing on a damaged spiking neural network with fixed output weights. Time courses of accuracy in the reservoir computing tasks for the six damaging conditions. The colors correspond to each damaging action. The training of the output layer stopped before the damage. Grey curves show the undamaged case for the tracks-patterned network, and the brown curves show the undamaged case for the control, unpatterned network. In the panels, the lines indicate the averaged value, and shadings the 95% confidence interval. *$p < 0.05$; **$p < 0.01$; ***$p < 0.001$ (two-sided unpaired t-test, $n = 15$, $df = 13$).
